# Comparison of the ELISA and ECL Assay for Vedolizumab Anti-drug Antibodies: Assessing the Impact on Pharmacokinetics and Safety Outcomes of the Phase 3 GEMINI Trials

**DOI:** 10.1208/s12248-020-00518-0

**Published:** 2020-11-16

**Authors:** Timothy Wyant, Lili Yang, Maria Rosario

**Affiliations:** 1 Present Address: Biolojic Inc., 100 Cambridge St., Cambridge, Massachusetts USA; 2 Takeda Pharmaceuticals International Inc., Cambridge, Massachusetts USA; 3 Development Center Americas Inc, Cambridge, Massachusetts USA

**Keywords:** electrochemiluminescence, ELISA, immunogenicity, vedolizumab

## Abstract

Vedolizumab immunogenicity has been assessed using an enzyme-linked immunosorbent assay (ELISA) with a ~ 0.5 μg/mL drug interference, which may underestimate on-drug immunogenicity. We aimed to compare immunogenicity results between ELISA and the new drug-tolerant electrochemiluminescence (ECL) assay (and the two versions of neutralizing assays, drug-sensitive versus drug-tolerant). The ECL assay drug tolerance is ~ 100 times higher than that of the ELISA (≥ 50 μg/mL *vs.* 0.5 μg/mL with a 500 ng/mL positive control), and assay sensitivity is < 5 ng/mL for both assays. Vedolizumab immunogenicity was assessed in 2000 GEMINI 1 and 2 patients originally tested by ELISA and retested by ECL assay. Anti-drug antibody (ADA) impact on infusion-related reactions and pharmacokinetics (PK) was examined using descriptive statistics and population PK analyses. By ECL assay, 6% (86/1427) of patients treated with vedolizumab as induction and maintenance therapy tested ADA-positive. Of these, 20 patients were persistently positive and 56 had neutralizing antibodies. By ELISA, 4% (56/1434) of these patients were ADA-positive, 9 were persistently positive, and 33 had neutralizing antibodies. Among 61 patients with infusion-related reactions, 6 (10%) were ADA-positive (2 persistently positive) by ECL assay. By ELISA, 3 (5%) patients were both ADA-positive and persistently positive. Most results (96%) were similar with both assays. In the updated population PK model, ADA-positive status was estimated to increase vedolizumab linear clearance by a factor of 1.10 (95% credible interval 1.03–1.17), which is consistent with previous reports. The impact of ADA on safety and PK modeling remained generally consistent using either ELISA or ECL assay. ClinicalTrials.gov: NCT00783718 and NCT00783692

## INTRODUCTION

Biologic therapies can trigger the formation of anti-drug antibodies (ADAs), an immune reaction identified as a contributor to loss of therapeutic response (1). As such, it is important to understand the extent to which biologic therapies induce an immunogenic response and whether immunogenicity has an adverse impact on pharmacokinetics (PK), safety, or efficacy.

Vedolizumab is a humanized recombinant monoclonal antibody that specifically binds to the α_4_β_7_ integrin and prevents the recruitment of α_4_β_7_ integrin leucocytes to the gut (2). Vedolizumab safety and efficacy are well established from multiple clinical (3,4) and real-world (5) studies, and it is approved for the treatment of moderately to severely active ulcerative colitis (UC) and Crohn’s disease (CD) (6–9). Pivotal vedolizumab phase 3, randomized, placebo-controlled, double-blind studies included GEMINI 1 (NCT00783718) in UC and GEMINI 2 (NCT00783692) and 3 (NCT01224171) in CD (3,4,10).

Vedolizumab immunogenicity was initially assessed in patients from GEMINI 1 and GEMINI 2 using an enzyme-linked immunosorbent assay (ELISA), which was state of the art at the time (11). Fifty-six of 1434 patients evaluated (4%) demonstrated vedolizumab ADA positivity at any time during the studies. Nine patients were considered persistently ADA-positive (defined as positive ADA in 2 or more consecutive samples), and 33 patients developed neutralizing antibodies (11). However, based on the assay validation, it was known that the presence of ~ 0.5 to 1 μg/mL vedolizumab with 500 ng/mL ADA-positive control in the serum interferes with the ELISA. As a result, it is possible that the on-drug immunogenicity rates may have been underestimated.

An acid dissociation electrochemiluminescence (ECL) ADA assay with a higher drug tolerance to detect ADAs in the presence of vedolizumab was developed and used to reassess vedolizumab immunogenicity in these patients. In addition, a more drug-tolerant ECL assay was developed to detect neutralizing ADAs to further characterize the confirmed positive ADA samples. Here, we present updated combined GEMINI 1 and GEMINI 2 vedolizumab immunogenicity results as assessed with improved ECL ADA assays and compare the magnitude of the impact on immunogenicity between the drug-tolerant and drug-sensitive assays.

## MATERIALS AND METHODS

### Immunogenicity Analyses

Vedolizumab ADAs were initially measured using an ELISA with a drug tolerance of 0.5 μg/mL vedolizumab at 500 ng/mL positive control (affinity purified rabbit anti-vedolizumab antibodies) and ≤ 20 μg/mL vedolizumab at 5 μg/mL positive control and assay sensitivity of 0.44 ng/mL. As an alternative to the traditional ELISA previously used to analyze vedolizumab immunogenicity (12,13), our team (Takeda Pharmaceuticals International Inc., Cambridge, MA) developed a new ADA assay using acid dissociation ECL. This new assay has a drug tolerance of at least 50 μg/mL vedolizumab with 500 ng/mL positive control, ≤ 25 μg/mL vedolizumab with 100 ng/mL positive control, and ≤ 5 μg/mL vedolizumab with 10 ng/mL positive control. Relative assay sensitivity using a positive control was 3.9 ng/mL, with inter- and intra-assay coefficients of variability for positive control and negative controls (a pooled human serum) < 25%. All available banked serum samples from GEMINI 1 and GEMINI 2 were tested for the presence of ADAs, followed by confirmation and titration in ECL and ELISA (Table I), and results were then compared between the two assays.

**Table I:** GEMINI 1 and GEMINI 2 Patients and Serum Samples Analyzed Using the ECL Assay and the ELISA

	GEMINI 1		GEMINI 2	
	ECL assay, tested/total	ELISA, tested/total	ECL assay, tested/total	ELISA, tested/total
Patients, *n*/*N*	893/895	894/895	1107/1115	1115/1115
Maintenance study ITT, *n*				
Placebo (from week 6)	126	126	152	153
Vedolizumab Q8W	122	122	154	154
Vedolizumab Q4W	125	125	154	154
Non-ITT, *n*				
Placebo (from week 0)	147	148	148	148
Vedolizumab Q4W (week 6 nonresponders)	373	373	499	506
Combined, *n*				
Continuous vedolizumab	620	620	807	814
Serum samples, *n*/*N*	4326^a^/4454	4454/4454	5180^b^/5420	5420/5420

*ECL electrochemiluminescence, ELISA enzyme-linked immunosorbent assay, ITT intent to treat, Q4W every 4 weeks, Q8W every 8 weeks*

^*a*^*Three serum samples did not have sufficient volume for analysis*

^*b*^*Two serum samples did not have sufficient volume for analysis*

Immunogenicity was evaluated by the ADA and neutralizing antibody status. Positive or negative ADA status was determined according to the definitions previously established to assess ELISA results, wherein samples that were positive in both the screening and confirmatory assays were designated as positive (14). ADA transient positive was defined as a patient who had tested ADA-positive at least once at any time during the treatment or post-treatment periods. ADA persistent positive was defined as a patient who had tested ADA-positive on at least two sequential time points during the treatment or post-treatment periods.

#### Detection of ADA by ELISA (Screening, Confirmation, and Titer Assessment)

A qualitative bridging assay format was used. The plate was pre-coated with vedolizumab, and the immobilized vedolizumab captured the ADA in serum samples, followed by biotinylated-vedolizumab and HRP-labeled streptavidin. When incubated with tetramethylbenzidine substrate, positive color development indicated the presence of ADAs in the samples.

#### Detection of Neutralizing ADA by a Cell-Based Assay

Samples confirmed ADA-positive by ELISA were subsequently analyzed for neutralizing ability against vedolizumab using the cell line RPM18866 (human myelogenous leukemia, lymphocyte B cell line) that expresses vedolizumab’s target α_4_β_7_ on the cell surface. In the absence of any neutralizing antibodies, addition of a biotinylated-vedolizumab resulted in specific binding of vedolizumab to the cell surface. This bound drug was detected by the addition of streptavidin-PE, which also binds to the bound biotinylated vedolizumab, and evaluated on a flow cytometer. Mean fluorescent intensity (MFI) expression correlated to the amount of bound drug on the cell surface. The presence of neutralizing antibodies in the patient serum prevented biotinylated vedolizumab from binding to the RPM18866 cell surface. A reduction of the MFI as compared to the MFI of negative control serum sample indicated neutralization.

#### Detection of ADA by ECL Assay (Screening, Confirmation, and Titer Assessment)

Patient serum samples, positive controls, and negative controls were first diluted and incubated with 300 mM acetic acid (pH 3), followed by a neutralization step containing 1 M TRIS buffer (pH 9.5), biotinylated vedolizumab, and SULFO-TAG-vedolizumab (SULFO-TAG is a product of Meso Scale Discovery). The samples were incubated on a polypropylene microtiter plate. During this time, ADA bound to both the biotinylated vedolizumab and SULFO-TAG-vedolizumab formed an antibody-drug complex bridge. After incubation, the mixture was added to the wells of a blocked streptavidin-coated MSD plate, which allowed biotin-drug-Ab-SULFO-TAG drug complexes to bind to the streptavidin-coated plate. After washing, tripropylamine-containing read buffer was added, and the chemiluminescent signal was measured in relative light unit by a SECTOR Imager 600/6000 instrument.

#### Detection of Neutralizing ADA by a Plate-Based Assay

Samples confirmed ADA-positive by ECL assay were subsequently analyzed in a new drug-tolerant neutralizing assay in which ADAs in patient serum samples were captured by biotinylated vedolizumab that was bound to a streptavidin-coated plate. After washing, the bound ADA was released with an acid dissociation step. The extracted ADAs were incubated with SULFO-TAG-vedolizumab before transferring to a α_4_β_7_-coated MSD plate. The tripropylamine-containing buffer was used for ECL signal detection. A decreased signal when compared with the negative control serum samples indicated the presence of neutralizing ADA.

### Study Design

GEMINI 1 was a phase 3, randomized, double-blind, placebo-controlled study consisting of separate induction and maintenance phases in patients with moderately to severely active UC (3). The induction phase included 2 patient cohorts: in cohort 1, patients were randomly assigned to receive vedolizumab or placebo, and cohort 2 received open-label vedolizumab. Patients from either cohort who demonstrated clinical response to vedolizumab at week 6 were re-randomized to receive vedolizumab every 8 weeks (Q8W) and every 4 weeks (Q4W) or placebo up to week 52 beginning at week 6 (maintenance intent-to-treat [ITT] population).

GEMINI 2 was a similarly designed trial in patients with moderately to severely active CD (4). Efficacy and safety outcomes have been previously reported (3,4). In GEMINI 1 and GEMINI 2, patient blood samples were collected at weeks 0 (predose), 6, 14, 26, 38, 52 (or early termination), and 66 (final safety visit for patients not enrolled in the extended access program) (15).

Patients were grouped based on vedolizumab treatment duration in GEMINI 1 and GEMINI 2. The first group included patients who never received vedolizumab (true placebo), that is, those who were randomized to placebo beginning at week 0 and did not receive vedolizumab in either the induction or maintenance phases (*n* = 297). The second group was the placebo (ITT) group, which consisted of patients who received only 2 doses of vedolizumab during the induction phase and were then randomized to receive placebo (*n* = 279). The third group consisted of patients who received vedolizumab up to week 52, that is, those who were maintained on open-label vedolizumab Q4W (*n* = 879), and from the ITT population who received vedolizumab Q8W (*n* = 276) or vedolizumab Q4W (*n* = 279) during maintenance. Overall, the combined vedolizumab group included the 1434 patients who received vedolizumab during both the induction and maintenance phases. Patients in GEMINI 1 and GEMINI 2 were monitored for acute infusion-related reactions during and after infusions. Adverse events assessed by the investigator as infusion-related reactions were recorded.

Vedolizumab has a half-life of ~ 25 days, and the week 66 time point was approximately 4.5 to 5 half-lives after the last dose of vedolizumab at week 50. Therefore, vedolizumab concentration was anticipated to be below the assay interference level, and this time point provided an opportunity to determine an “off-drug” rate for immunogenicity. Among patients with samples that had been previously analyzed by ELISA, samples from 2000 out of 2009 patients were available for reanalysis using the ECL assay (Table I).

### Population Pharmacokinetic Model Reanalysis

The impact of ADAs as a covariate that may affect clearance of vedolizumab was assessed using a previously reported population PK model (12) that was reanalyzed with the GEMINI 1 and GEMINI 2 immunogenicity samples. The population PK analysis of the repeated measures was conducted using a qualified installation of Nonlinear Mixed Effects Modelling (NONMEM®), version 7.3 (ICON Development Solutions, Hanover, MD). The previous final population PK model was fit to the data using the full Bayesian Markov Chain Monte Carlo (MCMC) method. The same Bayesian prior probability distributions that were specified in the previous model were used. In brief, informative priors were defined for maximum elimination rate (*V*_max_), concentration at half-maximum elimination rate (*K*_m_), peripheral volume of distribution (*V*_p_), and intercompartmental clearance (*Q*), whereas uninformative (vague) priors were defined for the remaining fixed-effect parameters (structural PK parameters and covariate coefficients) and interindividual random-effect parameters in the model. Covariates included body weight, age, sex, serum albumin, vedolizumab ADA status, fecal calprotectin, disease activity scores (Crohn’s Disease Activity Index and Mayo score, both partial and complete), prior anti-tumor necrosis factor (TNF)-α treatment, disease (CD and UC), and adjuvant therapy (methotrexate, azathioprine, 6-mercaptopurine, and aminosalicylates). Pre- and post-processing of model input/output and analysis scripting was programmed using version 3.2.3 of *R* (16).

## RESULTS

### ADA Status

Banked samples from 2000 patients treated with placebo for induction and maintenance (*n* = 295), vedolizumab for induction then placebo for maintenance (*n* = 278), or vedolizumab for both induction and maintenance (*n* = 1427) were reanalyzed in this study (Table I). ADA status using the new, more drug-tolerant ECL assay is presented in Table II, and the results obtained using the ELISA are presented in Table III. Among the 1427 patients who received vedolizumab continuously during induction and maintenance and had an immunogenicity sample available, 86 (6%) were ADA-positive with the ECL assay at any time during or after study treatment; 66 were transiently ADA-positive and 20 persistently ADA-positive. Fifty-six patients (4%) developed neutralizing antibodies (Table II). At week 66, 45 of 310 patients (15%) had positive ADAs.

**Table II:** GEMINI 1 and GEMINI 2 Patient Vedolizumab ADA Status Using the ECL Assay.

	**Maintenance study ITT**^**2a**^			**Non-ITT**		**Combined**
	**Placebo**^**b**^ **(from week 6)**	**Vedolizumab Q8W**	**Vedolizumab Q4W**	**Placebo**^**c**^ **(from week 0)**	**Vedolizumab Q4W (week 6 nonresponders)**	**Continuous vedolizumab**^**d**^
**ADA status up to week 52,** ***n*** **(%)**	***N*** **= 278**	***N*** **= 276**	***N*** **= 279**	***N*** **= 295**	***N*** **= 872**	***N*** **= 1427**
ADA negative	218 (78)	259 (94)	269 (96)	287 (97)	813 (92)	1341 (94)
ADA positive	60 (22)	17 (6)	10 (4)	8 (3)	59 (7)	86 (6)
Transiently positive	12	12	8	1	46	66
Persistently positive	48	5	2	7	13	20
Any neutralizing ADA positive^e^	46	10	4	3	42	56
**ADA status at week 66,**^**f**^ ***n*** **(%)**	***N*** **= 24**	***N*** **= 26**	***N*** **= 35**	***N*** **= 44**	***N*** **= 249**	***N*** **= 310**
ADA positive	3 (13)	5 (19)	3 (9)	0	37 (15)	45 (15)

*Not all patients had evaluable samples. N = total number of patients with samples tested in each treatment group. Proportions are based on nonmissing values*

*ADA vedolizumab anti-drug antibody, ECL electrochemiluminescence, ITT intent to treat, Q4W every 4 weeks, Q8W every 8 weeks*

^*a*^*Patients who were responders to vedolizumab induction at week 6 and were randomized into the maintenance-phase ITT population*

^*b*^*Patients who received vedolizumab during induction and were randomized to placebo at the maintenance phase*

^*c*^*Patients who were randomized to placebo at both the induction and maintenance phases*

^*d*^*All patients who received induction and maintenance vedolizumab, including week 6 responders who were randomized to vedolizumab in the maintenance phase plus patients who did not respond at week 6 and received maintenance vedolizumab Q4W*

^*e*^*Neutralizing ADA with no reportable titer was considered missing (i.e., no detectable positive neutralizing ADA was present in the sample)*

^*f*^*Week 66 was the final safety visit and approximately 4.5 to 5 half-lives after the last dose of vedolizumab; 310 samples were available for this analysis*

**Table III:** GEMINI 1 and GEMINI 2 Patient Vedolizumab ADA Status Using the ELISA

	**Maintenance study ITT**^**a**^			**Non-ITT**		**Combined**
	**Placebo**^**b**^ **(from week 6)**	**Vedolizumab Q8W**	**Vedolizumab Q4W**	**Placebo**^**c**^ **(from week 0)**	**Vedolizumab Q4W (week 6 nonresponders)**	**Continuous vedolizumab**^**d**^
**ADA status up to week 52,** ***n*** **(%)**	***N*** **= 279**	***N*** **= 276**	***N*** **= 279**	***N*** **= 296**	***N*** **= 879**	***N*** **= 1434**
ADA negative	234 (84)	268 (97)	276 (99)	288 (97)	834 (95)	1378 (96)
ADA positive	45 (16)	8 (3)	3 (1)	8 (3)	45 (5)	56 (4)
Transiently positive	14	6	3	3	38	47
Persistently positive	31	2	0	5	7	9
Any neutralizing ADA positive^e^	24	4	3	4	26	33
**ADA status at week 66,**^**f**^ ***n*** **(%)**	***N*** **= 24**	***N*** **= 26**	**N = 35**	***N*** **= 47**	***N*** **= 259**	***N*** **= 320**
ADA positive	2 (8)	4 (15)	1 (3)	0	27 (10)	32 (10)

*Not all patients had evaluable samples. N = total number of patients with samples tested in each treatment group. Proportions are based on nonmissing values*

*ADA vedolizumab anti-drug antibody, ELISA enzyme-linked immunosorbent assay, ITT intent to treat, Q4W every 4 weeks, Q8W every 8 weeks*

^*a*^*Patients who were responders to vedolizumab induction at week 6 and were randomized into the maintenance-phase ITT population*

^*b*^*Patients who received vedolizumab during induction and were randomized to placebo at the maintenance phase*

^*c*^*Patients who were randomized to placebo at both the induction and maintenance phases*

^*d*^*All patients who received induction and maintenance vedolizumab, including week 6 responders who were randomized to vedolizumab in the maintenance phase plus patients who did not respond at week 6 and received maintenance vedolizumab Q4W*

^*e*^*Samples positive for neutralizing ADAs with no reportable titer were considered missing (i.e., no detectable positive neutralizing ADA was present in the sample)*

^*f*^*Week 66 was the final safety visit and approximately 4.5 to 5 half-lives after the last dose of vedolizumab; 320 samples were available for this analysis*

In comparison, with the ELISA, 56 of 1434 patients (4%) who received vedolizumab continuously as part of GEMINI 1 or GEMINI 2 were ADA-positive. Of those 56 patients, 47 were transiently ADA-positive, 9 were persistently ADA-positive, and 33 were positive for neutralizing antibodies (Table III). At week 66, 32/320 (10%) patients with samples available for analysis were ADA-positive (15).

Similar to a previous report using the ELISA for ADA detection (13), patients who only received 2 doses of vedolizumab during induction and then placebo during maintenance phase had the highest incidence of ADA-positive samples (Tables II and III). Using the ECL assay, 60 of 278 (22%) patients were ADA-positive at any time during or after study treatment; 12 patients were transiently ADA-positive, 48 were persistently ADA-positive, and 46 (17%) were positive for neutralizing antibodies (Table II). In comparison, the ELISA detected ADA-positive samples in 45 of 279 (16%) patients, among whom 14 were transiently ADA-positive, 31 were persistently ADA-positive, and 24 were positive for neutralizing antibodies (Table III).

For patients (*n* = 1427) who had available results from both the ELISA and ECL assays, 1375 (96%) had similar ADA status, and 4% changed ADA status between the two assays (Table IV). Among the patients who showed differences in ADA status, 41 of 52 patients changed from negative by ELISA to positive by ECL assay, and 11 patients changed from positive by ELISA to negative by ECL assay. Of these 11 patients, 10 were transiently positive and only 1 was persistently positive by ELISA.

**Table IV:** Overall ADA Status for ELISA versus ECL Assay (continuous vedolizumab)a

	ECL	
ELISA	Negative	Positive
Negative	1330 (93)	*41 (3)*
Positive	*11 (1)*	45 (3)

*Only patients who had results from both the original and new assays are included*

*Italicized values indicate that ADA status changed between the ELISA and ECL assay*

*ADA anti-drug antibodies, ECL electrochemiluminescence, ELISA enzyme-linked immunosorbent assay, Q4W every 4 weeks*

^*a*^*All patients who received induction and maintenance vedolizumab, including week 6 responders who were randomized to vedolizumab in the maintenance phase plus patients who did not respond at week 6 and received maintenance vedolizumab Q4W*

Among the 1434 patients initially analyzed with ELISA and 1427 patients reanalyzed with ECL assay who were treated with vedolizumab during GEMINI 1 or GEMINI 2 induction and maintenance phases, 61 (4%) had an adverse event assessed by the investigator as an infusion-related reaction. With the ECL essay, 6 (10%) of these patients were ADA-positive, with 2 persistently ADA-positive (Table V). In comparison, with the ELISA, 3 of 61 (5%) patients were ADA-positive and all 3 were persistently ADA-positive (Table VI).

**Table V:** Infusion-Related Reactions and Immunogenicity Status of Patients During Vedolizumab Maintenance in GEMINI 1 and GEMINI 2 Using the ECL Assay

	Maintenance study ITT^a^			Non-ITT		Combined
AEs defined by the investigator as infusion-related reactions (yes/no), *n* (%)	Placebo^b^ (from week 6)	Vedolizumab Q8W	Vedolizumab Q4W	Placebo^c^ (from week 0)	Vedolizumab Q4W (week 6 nonresponders)	Continuous vedolizumab^d^
	*N* = 278	*N* = 276	*N* = 279	*N* = 295	*N* = 872	*N* = 1427
Yes	8	12	18	9	31	61
ADA negative	5 (63)	9 (75)	18 (100)	9 (100)	28 (90)	55 (90)
ADA positive	3 (38)	3 (25)	0	0	3 (10)	6 (10)
Transiently positive	1	2	0	0	2	4
Persistently positive	2	1	0	0	1	2
Any neutralizing ADA positive^e^	2	3	0	0	2	5
No	270	264	261	286	841	1366
ADA negative	213 (79)	250 (95)	251 (96)	278 (97)	785 (93)	1286 (94)
ADA positive	57 (21)	14 (5)	10 (4)	8 (3)	56 (7)	80 (6)
Transiently positive	11	10	8	1	44	62
Persistently positive	46	4	2	7	12	18
Any neutralizing ADA positive^e^	44	7	4	3	40	51

*Not all patients had evaluable samples. N = total number of patients with samples tested in each treatment group. Proportions are based on nonmissing values.*

*AE adverse event, ADA vedolizumab anti-drug antibody, ECL electrochemiluminescence, ITT intent to treat, Q4W every 4 weeks, Q8W every 8 weeks*

^*a*^*Patients who were responders to vedolizumab induction at week 6 and were randomized into the maintenance-phase ITT population*

^*b*^*Patients who received vedolizumab during induction and were randomized to placebo at the maintenance phase*

^*c*^*Patients who were randomized to placebo at both the induction and maintenance phases*

^*d*^*All patients who received induction and maintenance vedolizumab, including week 6 responders who were randomized to vedolizumab in the maintenance phase plus patients who did not respond at week 6 and received maintenance vedolizumab Q4W*

^*e*^*Samples positive for neutralizing ADAs with no reportable titer were considered missing (i.e., no detectable positive neutralizing ADA was present in the sample)*

**Table VI:** Infusion-Related Reactions and Immunogenicity Status of Patients During Vedolizumab Maintenance in GEMINI 1 and GEMINI 2 Using ELISA

AEs defined by the investigator as infusion-related reactions (yes/no), *n* (%)	Maintenance study ITT^a^			Non-ITT		Combined
	Placebo^b^ (from week 6)	Vedolizumab Q8W	Vedolizumab Q4W	Placebo^c^ (from week 0)	Vedolizumab Q4W (week 6 nonresponders)	Continuous vedolizumab^d^
	*N* = 279	*N* = 276	*N* = 279	*N* = 296	*N* = 879	*N* = 1434
Yes	8	12	18	9	31	61
ADA negative	7 (88)	11 (92)	18 (100)	9 (100)	29 (94)	58 (95)
ADA positive	1 (13)	1 (8)	0	0	2 (6)	3 (5)
Transiently positive	0	0	0	0	0	0
Persistently positive	1	1	0	0	2	3
Any neutralizing ADA positive^e^	0	0	0	0	2	2
No	271	264	261	287	848	1373
ADA negative	227 (84)	257 (97)	258 (99)	279 (97)	805 (95)	1320 (96)
ADA positive	44 (16)	7 (3)	3 (1)	8 (3)	43 (5)	53 (4)
Transiently positive	14	6	3	3	38	47
Persistently positive	30	1	0	5	5	6
Any neutralizing ADA positive^e^	24	4	3	4	24	31

*Not all patients had evaluable samples. N = total number of patients with samples tested in each treatment group. Proportions are based on nonmissing values*

*AE adverse event, ADA vedolizumab anti-drug antibody, ELISA enzyme-linked immunosorbent assay, ITT intent to treat, Q4W every 4 weeks, Q8W every 8 weeks*

^*a*^*Patients who were responders to vedolizumab induction at week 6 and were randomized into the maintenance-phase ITT population*

^*b*^*Patients who received vedolizumab during induction and were randomized to placebo at the maintenance phase*

^*c*^*Patients who were randomized to placebo at both the induction and maintenance phases*

^*d*^*All patients who received induction and maintenance vedolizumab, including week 6 responders who were randomized to vedolizumab in the maintenance phase plus patients who did not respond at week 6 and received maintenance vedolizumab Q4W*

^*e*^*Samples positive for neutralizing ADA with no reportable titer were considered missing (i.e., no detectable positive neutralizing ADA was present in the sample)*

Of the 278 patients who received 2 doses of vedolizumab during the induction phase then placebo during the maintenance phase and tested with the ECL assay, 8 (3%) had infusion-related reactions, with 3 of the 8 (38%) ADA-positive, and 2 persistently-positive (Table V). In comparison, of the 279 patients tested with ELISA, only 1 of 8 (13%) was determined to be ADA-positive (Table VI).

### Vedolizumab Pharmacokinetics

It was previously reported that the presence of ADA is associated with lower serum concentrations of vedolizumab (13). Therefore, trough serum vedolizumab concentrations were determined along with ADA status in 1713 patients who received at least 1 dose of vedolizumab in GEMINI 1 or GEMINI 2. At week 52, the median serum vedolizumab trough concentrations were 20.5 μg/mL, 18.4 μg/mL, and below the limit of quantitation for ADA-negative, transiently ADA-positive, and persistently ADA-positive patients with UC, respectively. For ADA-negative, transiently ADA-positive, and persistently ADA-positive patients with CD, the medians were 18.7 μg/mL, 9.6 μg/mL, and below the limit of quantitation, respectively. The drug tolerance of ADA ECL assay using the 100 ng/mL surrogate ADA-positive control is 25 μg/mL, which is greater than the median trough concentrations of vedolizumab observed in clinical trials. Therefore, the assay was able to detect the clinically relevant ADA as suggested by the FDA 2019 guidance (17).

### Population PK Model Reanalysis

Samples from all patients who received vedolizumab at any time during GEMINI 1 or GEMINI 2 were used to update the vedolizumab population PK model (12). The median age of the reanalysis patient population was 35.9 years of age (range, 17.7–77.7 years) and 48% were female. Patients had a median body weight of 68.3 kg (range, 28.0–172 kg), median albumin concentrations of 3.70 g/dL (range, 1.40–5.30 g/dL), and median fecal calprotectin concentrations of 731 mg/kg (range, 23.8–2.00e^4^ mg/kg). The median partial Mayo score of the 743 (43%) patients with UC was 6.0 (range, 1.0–9.0), and the median Crohn’s Disease Activity Index score for the 966 (57%) patients with CD was 321 (range, 93.0–548.0).

As previously reported, body weight and serum albumin had an effect on vedolizumab linear clearance (CL_L_) variability with the potential to be clinically relevant (12). Also consistent with previous results obtained with ELISA showing an increase in CL_L_ of 1.12 (95% credible interval (CDI), 1.05–1.20), the presence of ADA detected by the ECL assay was estimated to increase vedolizumab CL_L_ by a factor of 1.10 (95% CDI, 1.03–1.17) (Table VII). With both models, the 95% CDI was statistically different from the null effect of 1. Further evaluation of covariate effects on CL_L_ was conducted via simulation given Bayesian joint posterior distribution (or uncertainty) of the model parameters.

**Table VII:** Covariate Parameters Estimated from the Final Population Pharmacokinetic Model Using Data from Both the ECL Assay and the ELISA

Parameter	ECL		ELISA	
	Estimate	Bayesian 95% CDI	Estimate	Bayesian 95% CDI
Continuous covariates^a^				
CL_L_~weight	0.339	(0.264 to 0.406)	0.368	(0.306 to 0.433)
CL_L_~albumin	− 1.03	(− 1.12 to − 0.940)	− 1.18	(− 1.24 to − 1.13)
CL_L_~fecal calprotectin	0.0279	(0.0204 to 0.0349)	0.0312	(0.0257 to 0.0368)
CD CL_L_~CDAI	− 0.0582	(− 0.151 to 0.0337)	− 0.0558	(− 0.144 to 0.0311)
UC CL_L_~partial Mayo score	0.0543	(− 0.0227 to 0.132)	0.0406	(− 0.0339 to 0.115)
CL_L_~age	− 0.0190	(− 0.0673 to 0.0294)	− 0.0339	(− 0.0778 to 0.0103)
*V*_c_~weight	0.456	(0.409 to 0.502)	0.469	(0.427 to 0.511)
*V*_p_~weight	1.00 fixed	—	1.00 fixed	(1.00 to 1.00)
*V*_max_~weight	0.750 fixed	—	0.75 fixed	(0.75 to 0.75)
*Q*~weight	0.750 fixed	—	0.75 fixed	(0.75 to 0.72)
Categorical covariates^b^				
CL_L_~TNF	1.05	(1.01 to 1.09)	1.04	(1.01 to 1.07)
CL_L_~ADASUB	1.10	(1.03 to 1.17)	1.12	(1.05 to 1.2)
CL_L_~AZA full duration	0.998	(0.960 to 1.04)	0.992	(0.958 to 1.03)
CL_L_~AZA unknown duration	0.963	(0.876 to 1.05)	0.965	(0.886 to 1.05)
CL_L_~MP full duration	1.05	(0.949 to 1.17)	1.07	(0.97 to 1.18)
CL_L_~MP unknown duration	1.12	(0.989 to 1.26)	1.09	(0.974 to 1.22)
CL_L_~MTX full duration	1.02	(0.923 to 1.12)	1.02	(0.933 to 1.11)
CL_L_~MTX unknown duration	1.02	(0.871 to 1.20)	0.951	(0.825 to 1.09)
CL_L_~AMINO full duration	1.01	(0.969 to 1.05)	1.02	(0.984 to 1.06)
CL_L_~AMINO unknown duration	0.959	(0.903 to 1.02)	0.972	(0.922 to 1.02)
*V*_c_~diagnosis (CD or UC)	1.01	(0.985 to 1.04)	1.01	(0.989 to 1.03)

*AMINO aminosalicylate adjuvant therapy, ADA vedolizumab anti-drug antibody, ADASUB patient-level ADA incidence indicator, AZA azathioprine adjuvant therapy, CD Crohn’s disease, CDAI Crohn’s Disease Activity Index, CDI credible interval, CL*_*L*_ *linear clearance, ECL electrochemiluminescence, ELISA enzyme-linked immunosorbent assay, MP mercaptopurine adjuvant therapy, MTX methotrexate adjuvant therapy, Q intercompartmental clearance, TNF tumor necrosis factor, UC, ulcerative colitis, V*_*c*_ *central compartment volume, V*_*p*_ *peripheral compartment volume, V*_*max*_ *maximum elimination rate*

^*a*^*Null effect = 0*

^*b*^*Null effect = 1*

**Fig. 1:**
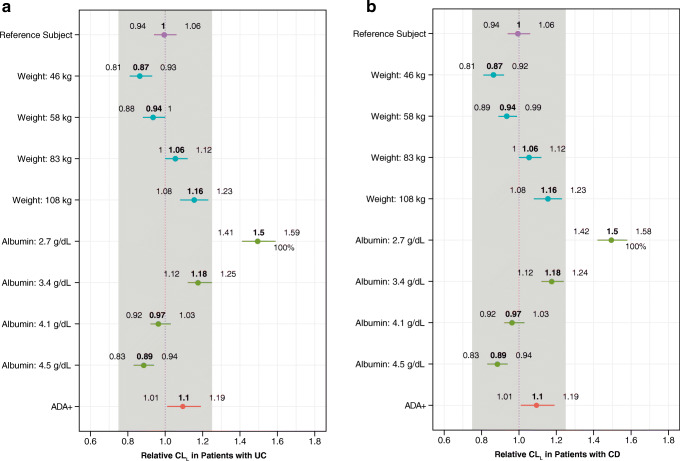
Modeled effect of covariates on vedolizumab linear clearance (CL_L_) using the ECL assay data. CL_L_ for **a** patients with UC and **b** patients with CD relative to a typical reference patient (UC referent 70 kg, albumin 4 g/dL, fecal calprotectin 700 mg/kg, partial Mayo score 6, 40 years old, naive anti-TNFα therapy, ADASUB-negative, and no adjuvant azathioprine, mercaptopurine, methotrexate, or aminosalicylate therapy; CD referent 70 kg, albumin 4 g/dL, fecal calprotectin 700 mg/kg, CDAI score 300, 40 years old, anti-TNF therapy naive, ADA-negative, and no adjuvant azathioprine, mercaptopurine, methotrexate, or aminosalicylate therapy) is plotted by covariate value. Covariates were fixed to the reference values except when they were the subject of perturbation. Body weight and albumin were evaluated at the observed 5th, 25th, 75th, and 95th percentiles in the data set. The closed circles represent the median and the horizontal lines represent the derived 95% CDI. The vertical dashed line at *x* = 1 represents the typical reference patient, and the grey-shaded region represents a parameter change of ± 25% from the reference value of 1 (null effect). ADA vedolizumab anti-drug antibody, ADASUB patient-level vedolizumab ADA incidence indicator, CD Crohn’s disease, CDAI Crohn’s Disease Activity Index, CDI credible interval, ECL electrochemiluminescence, TNF tumor necrosis factor alpha, UC ulcerative colitis

The effect of covariates on vedolizumab CL_L_ by disease state is shown in Fig. 1. Covariate sizes of ± 25% from the typical reference subject were used as a limit for clinically meaningful changes (12). Although there was a trend toward greater CL_L_ with increased body weight for both the UC and CD cohorts, there was no clinically meaningful impact across the range of values evaluated. Similar observations were made for albumin except at the lowest value (median CL_L_, 1.5 [range, 1.41–1.59] and median CL_L_, 1.5 [range, 1.42–1.58] for both UC and CD, respectively). ADA positivity was not associated with a clinically significant change in CL_L_ in either UC or CD patients, (the entire 95% CDI for the covariate effect fell within the ± 25% limits).

## DISCUSSION

We report the results of a reanalysis of vedolizumab immunogenicity using new, more drug-tolerant, ECL-based assays for detection of anti-drug and neutralizing anti-drug antibodies. The availability of banked patient serum samples from the GEMINI 1 and GEMINI 2 trials offered a valuable opportunity to evaluate the new ECL assays on a large scale, compare the ECL results with those obtained using the traditional ELISA method, and reevaluate the effect of immunogenicity on vedolizumab PK and safety in inflammatory bowel disease.

The acid dissociation ECL assay used in this study has a drug tolerance at least 100 times higher than the previous ELISA when using a 500 ng/mL surrogate ADA-positive control, thus allowing for detection of low-titer ADA. One limitation of this ECL assay is that due to its drug tolerance of ≤ 25 μg/mL of vedolizumab using a 100 ng/mL positive control, the presence of vedolizumab trough concentrations higher than 25 μg/mL may interfere with the detection of low-titer ADA (10 ng/mL). Patients may have a drug trough concentrations of > 25 μg/mL during the induction phase of vedolizumab treatment. However, with the approved vedolizumab Q8W IV maintenance dosing schedule, patients are not expected to exceed drug trough concentrations of 25 μg/mL (6). Furthermore, the low positive control ADA concentration is 10-fold below the FDA-recommended levels of sensitivity and is likely not clinically relevant (17,18). Since vedolizumab is a humanized monoclonal antibody with no endogenous counterpart and it is not agonistic in function (14,19), this concentration poses a minimal risk to patient safety.

The presence of ADAs and neutralizing ADAs was assessed in a cohort of 1427 patients with UC and CD treated with vedolizumab for up to 52 weeks. In this reanalysis of vedolizumab immunogenicity, the rate of ADAs in patients who received maintenance vedolizumab remained low with the ECL assay (6%) and with the ELISA (4%). With the new ECL assay, the rate of ADA positivity continued to be generally low across all subgroups. Rates ranged from 3% in patients who were randomized to placebo at both the induction and maintenance phases to 22% in patients who received vedolizumab during induction and were randomized to placebo at the maintenance phase, which was slightly higher than what was detected with the ELISA (range, 3%–16%). Of interest, the “off-drug” rate of immunogenicity at week 66 was 15% in the vedolizumab combined group by the ECL assay compared with 10% using the ELISA (15), suggesting the increased drug tolerance of the ECL assay was able to identify additional positive samples. The ECL assay also detected a higher proportion of samples with neutralizing ADAs.

Both the ECL assay and the ELISA showed the highest incidence of ADA-positive individuals among patients who received vedolizumab at induction and then switched to placebo for maintenance. The reason for this observation is currently unknown. An elevated ADA-positivity rate following a drug holiday has been consistently observed with vedolizumab and other biologics used to treat inflammatory bowel disease (3,4,20,21). It has been suggested that this may be a result of the presence of less drug to interfere with ADA assays resulting in a better detection rate. However, the observed similar ADA incident rate by the more drug-tolerant ECL assay suggests factors other than drug interference might have contributed to this observation. Previous reports showed that higher ADA levels with episodic or interrupted biologic treatment as compared with continuous treatment are accompanied by decreased treatment benefit (21–24). Some reports suggested lower immunogenicity in the continuous treatment groups due to the induction of immunotolerance by maintaining a trough vedolizumab concentration, or the paradoxical suppression of the immune response (25,26).

The ADA status of most (96%) patients remained unchanged between ELISA and the ECL assay. Changing from ADA-negative by ELISA to ADA-positive by ECL assay was most likely due to improved assay drug tolerance, while changing from positive by ELISA to negative by ECL was most likely due to bioanalytical assay variation in detecting lower levels of antibody titers near the assay cut point. These results demonstrate that the two assays have general agreement but may differ in the presence of low levels of ADA.

Infusion-related reactions were infrequent in the GEMINI studies and were reported in only 61 out of 1434 patients initially analyzed with ELISA and 1427 patients reanalyzed with ECL assay who received vedolizumab maintenance treatment. While ADAs were detected in more patients with infusion reactions using the ECL assay than with the ELISA, the overall rate remained low (10% versus 5%), suggesting that immunogenicity is not a large driver of infusion reactions with vedolizumab.

Based on week 52 median vedolizumab trough concentrations, persistent ADA-positive antibodies as detected using both the ECL assay and the ELISA were associated with decreased vedolizumab serum concentrations. The results of vedolizumab CL_L_ covariate analyses were consistent with previous reports (12) and showed a trend for body weight and serum albumin to affect CL_L_, although the difference only reached clinical relevance (± 25%) at the lowest albumin concentration. Similar to ADA outcomes generated with the ELISA, the presence of ADAs as detected using the ECL assay was determined to be not clinically relevant, as the entire 95% CDI fell within the predetermined ± 25% limits.

## CONCLUSION

In conclusion, this re-analysis of vedolizumab immunogenicity in serum samples from a very large cohort of patients with UC or CD confirmed that immunogenicity results obtained using new, drug-tolerant ECL assays remain generally consistent with those obtained using ELISA. While the ECL assays detected slightly more ADA-positive patients than did the ELISA, these new ECL results support the conclusions established with ELISA that vedolizumab immunogenicity is low and has a minimal effect on vedolizumab PK or safety in patients with inflammatory bowel disease.
